# Large-scale assessment of lepidopteran soybean pests and efficacy of Cry1Ac soybean in Brazil

**DOI:** 10.1038/s41598-021-95483-9

**Published:** 2021-08-05

**Authors:** Renato J. Horikoshi, Patrick M. Dourado, Geraldo U. Berger, Davi de S. Fernandes, Celso Omoto, Alan Willse, Samuel Martinelli, Graham P. Head, Alberto S. Corrêa

**Affiliations:** 1 Bayer Crop Science, São Paulo, SP Brazil; 2 Departamento de Entomologia e Acarologia, Escola Superior de Agricultura “Luiz de Queiroz”, Universidade de São Paulo, Piracicaba, SP Brazil; 3 Regulatory Science, Bayer Crop Science, Chesterfield, MO 63017 USA

**Keywords:** Entomology, Environmental economics

## Abstract

The soybean technology MON 87701 × MON 89788, expressing Cry1Ac and conferring tolerance to glyphosate, has been widely adopted in Brazil since 2013. However, pest shifts or resistance evolution could reduce the benefits of this technology. To assess Cry1Ac soybean performance and understand the composition of lepidopteran pest species attacking soybeans, we implemented large-scale sampling of larvae on commercial soybean fields during the 2019 and 2020 crop seasons to compare with data collected prior to the introduction of Cry1Ac soybeans. *Chrysodeixis includens* was the main lepidopteran pest in non-*Bt* fields. More than 98% of larvae found in Cry1Ac soybean were *Spodoptera* spp., although the numbers of *Spodoptera* were similar between Cry1Ac soybean and non-*Bt* fields. Cry1Ac soybean provided a high level of protection against *Anticarsia gemmatalis*, *C. includens*, *Chloridea virescens* and *Helicoverpa* spp. Significant reductions in insecticide sprays for lepidopteran control in soybean were observed from 2012 to 2019. Our study showed that *C. includens* and *A. gemmatalis* continue to be primary lepidopteran pests of soybean in Brazil and that Cry1Ac soybean continues to effectively manage the target lepidopteran pests. However, there was an increase in the relative abundance of non-target *Spodoptera* spp. larvae in both non-*Bt* and Cry1Ac soybeans.

## Introduction

Brazil is a top producer of soybean (*Glycine max* (L.) Merrill)^1^, and a turning point in the commercial cultivation of soybean in Brazil was the expansion of soybean production from the South (subtropical climate) into areas in the savannahs (i.e., Cerrado) in the Central-West region of the country (tropical climate)^2^. This expansion was largely enabled by technological advances ranging from better soil management and fertilization practices to the development of soybean varieties adapted to equatorial latitudes^3^. Moreover, the expansion of no-till systems, the use of better planting and harvesting equipment, and the adoption of transgenic soybeans to assist in weed control made important contributions to increases in the national average soybean yield in Brazil^1,3–5^. As soybean production in Brazil transformed into a highly structured and organized large-scale business operation primarily targeting export markets, the need to adopt simple, low-cost agronomic practices for controlling insects caused an increase in the number of insecticide sprays required^6–8^.

In this context, the soybean technology MON 87701 × MON 89788 (Intacta RR2 PRO®), expressing the Cry1Ac insecticidal protein (event MON 87701) and conferring tolerance to glyphosate (event MON 89788), was commercially launched and became available to farmers in Brazil in 2013. The adoption and use of Cry1Ac soybean by Brazilian farmers increased from 1.2 million hectares in the 2013/14 cropping season to 21.9 million hectares in the 2017/18 cropping season^9^. The acceptance of this soybean technology by Brazilian farmers can be attributed to the cost-effective and simpler weed control enabled by the tolerance to glyphosate, coupled with higher yields from a combination of better pest and weed management^10^. Cry1Ac soybean provides high-level protection against the major soybean lepidopteran pests, including *Anticarsia gemmatalis* (Hübner, 1818) (Lepidoptera: Erebidae), *Chrysodeixis includens* (Walker [1858]) (Lepidoptera: Noctuidae), *Chloridea virescens* (Fabricius, 1777) (Lepidoptera: Noctuidae) and *Helicoverpa armigera* (Hübner, 1808) (Lepidoptera: Noctuidae)^11–14^. Despite its benefits to soybean pest management, Cry1Ac soybean does not confer protection against the main species of *Spodoptera* found in Brazil*: Spodoptera frugiperda* (J.E. Smith, 1797) (Lepidoptera: Noctuidae), *Spodoptera eridania* (Stoll, 1782) (Lepidoptera: Noctuidae) and *Spodoptera cosmioides* (Walker, 1898) (Lepidoptera: Noctuidae)^15^.

Beyond the direct benefit of controlling target pests, *Bt* crops such as Cry1Ac soybean have the potential to provide additional benefits to insect management in agricultural systems, including reduction in insecticide use^10^, compatibility with biocontrol measures^16,17^, and regional suppression of insect pest populations^18–21^. In particular, suppression of target pests after a long period of use of highly efficacious *Bt* technologies has been documented in *Pectinophora gossypiella* (Saund., 1844) (Lepidoptera: Gelechiidae), *Ostrinia nubilalis* (Hübner, 1796) (Lepidoptera: Pyralidae) and *Helicoverpa zea* (Boddie, 1850) (Lepidoptera: Noctuidae) in the USA^18,19,21^ and *H. armigera* in China^20^. Similarly, high efficacy of Cry1Ac soybean against lepidopteran pests such as *C. includens* and *A. gemmatalis*, the main soybean pests in Brazil^12^, resulted in fewer insecticide sprays to manage lepidopteran larvae after five years of commercial use in Brazil^10^. However, where a *Bt* technology is ineffective against non-target secondary pest species and or broad-spectrum insecticide use has decreased due to highly effective control of the target species, secondary pests may increase in abundance over time^22,23^.

In addition, resistance evolution by target pest populations can reduce the benefits of *Bt* crops^24^. The high-dose expression and refuge strategy was proposed to manage resistance of target pest populations to Cry1Ac soybean in Brazil^12^. Nevertheless, poor compliance with refuge recommendations has been a common factor in most cases of documented field-evolved resistance to *Bt* crops^25–29^. Moreover, the intensive use of agricultural land creates an environment conducive to the buildup of relatively large insect pest populations and multiple generations of pests potentially under selection of *Bt* crops or insecticides^30,31^. “Tropical agriculture” such as that practiced in Brazil is typically based on two or more crop seasons per year, allowing pest populations to go through multiple generations per year on *Bt* crops and consequently increasing selection pressure^30^. Therefore, understanding the performance of a *Bt* crop against target pests at the field level and determining whether non-target pests are increasing in abundance can inform the need for the adoption of appropriate Integrated Pest Management (IPM) practices in Brazil.

In this study, we carried out a two-year large-scale assessment on commercial soybean fields in Brazil after eight years of Cry1Ac soybean use with the goals of (a) evaluating Cry1Ac soybean performance and impacts on soybean pest management and (b) assessing the relative abundance of lepidopteran pest species attacking soybean fields and comparing these results to data collected prior to the commercial introduction of Cry1Ac soybeans.

## Methods

### Insect sampling and data collection

All insect collections were in accordance with the approval granted by the System of Authorization and Information on Biodiversity (SISBIO) of the Ministry of Environment to a contracted company responsible for the field sampling (PROMIP, Permit for scientific purpose activity: 61826, 61824).

#### Sampling prior to commercialization of Cry1Ac soybean

From 2011 (2010/11) to 2014 (2013/14), field sampling of lepidopteran larvae was carried out in plots of non-*Bt* (Roundup Ready—RR) soybean. Samples consisted of 10 beat sheets (length = 1 m) per location followed by identification of larvae. A total of 829 samples were taken across the states of Bahia (BA), Distrito Federal (DF), Goiás (GO), Mato Grosso (MT), Mato Grosso do Sul (MS), Minas Gerais (MG), Paraná (PR), Rio Grande do Sul (RS), Santa Catarina (SC), São Paulo (SP) and Tocantins (TO). These data from 2011–2014 provided a baseline for descriptive comparisons with the subsequent sampling described below but were not analyzed statistically.

#### Sampling after commercialization of Cry1Ac soybean

**Figure 1:**
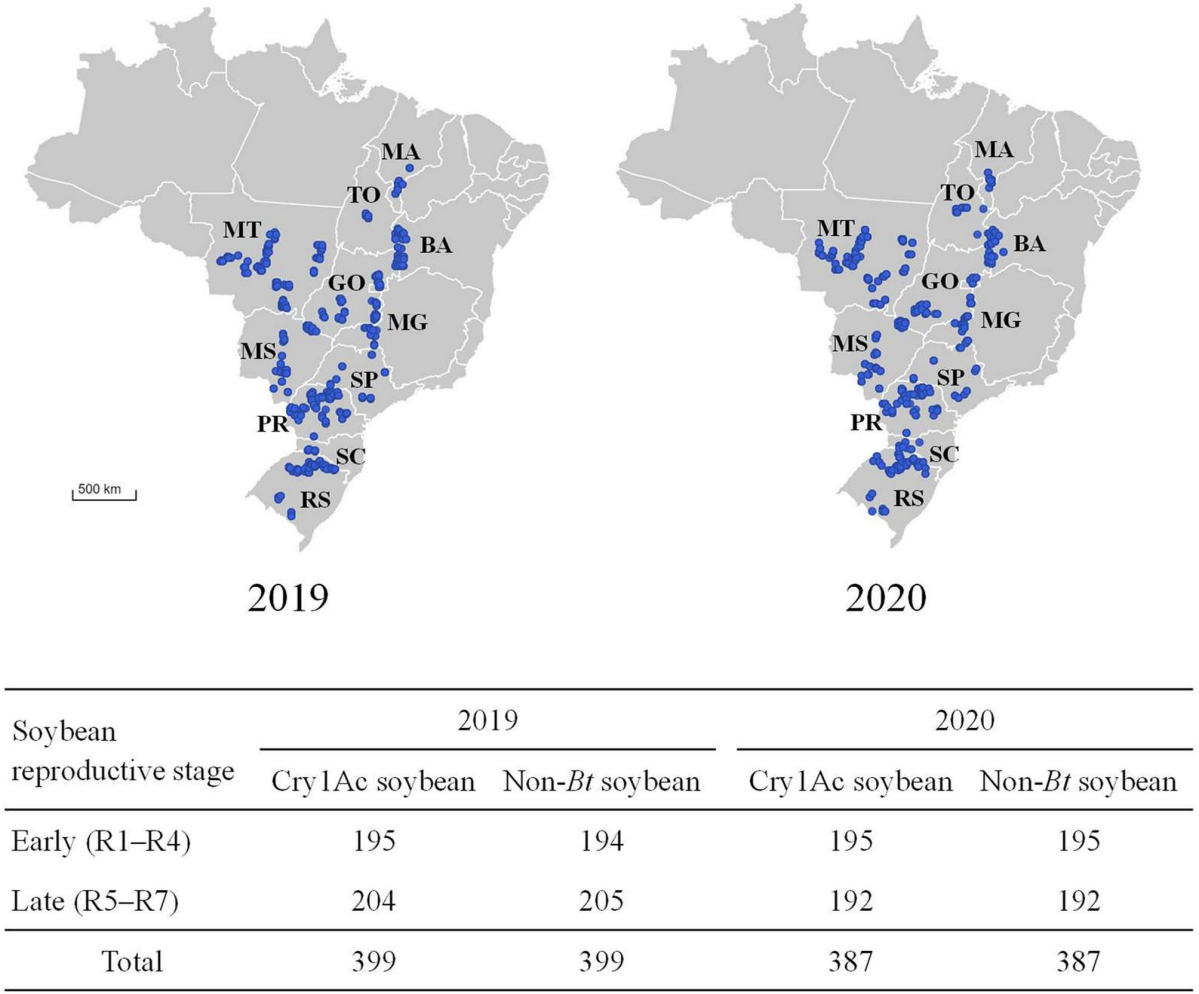
Locations and number of lepidopteran larvae samplings in commercial soybean fields in 2019 and 2020.

Lepidopteran larvae were sampled from commercial 399 soybean fields during the 2019 (2018/19) and 387 fields in 2020 (2019/20) cropping seasons (Fig. 1). Each location had a non-*Bt* (Roundup Ready—RR) soybean field and a Cry1Ac soybean (MON 87701 × MON 89788, Intacta RR2 PRO®) field. Samplings were conducted at early reproductive stages (R1–R4) and late reproductive stages (R5–R7). Larvae were sampled with a beat sheet (length = 1 m) and the sampling unit consisted of 10 beats in a zig-zag pattern per soybean reproductive stages. Additionally, for each beat sheet sampling, the level of defoliation in soybean was evaluated. To avoid border effects, sampling was initiated at a minimum of 20 m from the edge of the soybean fields in the Southern region of Brazil, where farms are smaller (average size of farms less than 100 ha), and 100 m in the Central-West and Northeast regions, where larger farms are common (average size of farms greater than 150 ha). For each location, sampling was done first in the non-*Bt* field: if at least 1 larva per meter was obtained, then samples were also taken from a nearby Cry1Ac soybean field at a similar plant growth stage to have a pair of neighboring fields with comparable incidence of lepidopteran pests. All fields were checked for Cry1Ac expression using QuickStix kits (Envirologix) to confirm the presence of *Bt* protein in Cry1Ac soybean plants and absence of this protein in non-*Bt* soybean plants. Larvae were transferred to 50-mL labeled conical centrifuge tubes containing propylene glycol. All tubes were then sent to the laboratory and kept in a freezer (− 20 °C) until identification. All lepidopteran larvae were identified based on Herzog^32^, Sosa-Gómez et al.^33^ and Gilligan and Passoa^34^.

### Lepidopteran species composition sampled from non-*Bt* fields prior and after commercialization of Cry1Ac soybean

A descriptive analysis was made with the total insects sampled prior (2011–2014) and after (2019–2020) commercialization of Cry1Ac soybean. The relative number of each species for 2011–14 and 2019–20 was represented as a percentage of total.

### Comparison of pest abundance on Cry1Ac soybean and non-*Bt* soybean

Fields with Cry1Ac soybean were paired with neighboring non-*Bt* fields (see “Insect sampling and data collection”). To compare larval counts in Cry1Ac soybean fields with counts in non-*Bt* soybean fields, a generalized linear mixed model with Poisson link was fit to the data separately for each species and plant growth stage (combined across seasons 2019 and 2020). If ${Y}_{ij}$ is the count for the *i*th field type in the *j*th pair, then $E\left(\mathrm{log}\left({Y}_{ij}\right)\right)={u}_{j}$ for non-*Bt* fields, and $E\left(\mathrm{log}\left({Y}_{ij}\right)\right)={\tau }{+u}_{j}$ for Cry1Ac soybean fields, where ${u}_{j}\sim N\left(0,{\sigma }^{2}\right)$ is the effect of the *j*th pair and $\mathrm{exp}(\tau )$ is the relative larvae count in Cry1Ac soybean fields. Analyses were performed with R statistical software—R version 4.0.2^35^.

### Pest species contributions to defoliation

To determine the relative contribution of individual species to defoliation, multiple regression was performed of defoliation against counts of *A. gemmatalis*, *C. includens*, *C. virescens*, *Helicoverpa* spp., *Rachiplusia nu* (Guenée) (Lepidoptera: Noctuidae), *S. cosmioides*, *S. eridania* and *S. frugiperda*. Regression was conducted separately for Cry1Ac soybean and non-*Bt* soybean, and for early and late growth reproductive stages, combined across the 2019 and 2020 seasons. The regression coefficient for a given species can be interpreted as the percentage increase in defoliation for each individual larva present. Thus, species with large coefficients contributed more to defoliation than did species with small coefficients. Analyses were performed with R statistical software—R version 4.0.2^35^.

### Geographic variation in soybean pest abundance

Sampling locations were grouped according to Embrapa’s soybean variety regionalization^36^. These groupings are called “edaphoclimatic regions” and “soybean macroregions” and are based on agroecological zones, Köppen climate classification for Brazil, technical recommendations for soybean production, soybean research meeting documents, and contributions of research institutes^36^. To characterize geographic variation in pest abundance, random effects for edaphoclimatic regions were estimated using a linear mixed-effects model for larval count data with Poisson link, with edaphoclimatic region nested within soybean macroregion. The abundance was estimated based on non-*Bt* soybean larval sampling. The edaphoclimatic region estimates were color-coded in choropleth maps. Analyses were performed with R statistical software—R version 4.0.2^35^.

### Insecticide use on soybean fields in Brazil

Data on use of insecticide sprays to manage lepidopteran larvae across mesoregions for the 2013 to 2019 cropping seasons were obtained from the AMIS Kleffmann Group database (2013–2018) and BIP Spark (2019). A linear regression analysis was performed with number of insecticide sprays as a function of cropping season. The number of insecticide sprays for lepidopteran control in every mesoregion of soybean planting area was log-transformed. Analyses were performed in GraphPad Prism 8 (GraphPad Software, San Diego, CA, USA).

## Results

### Lepidopteran species composition sampled from non-*Bt* fields

**Figure 2:**
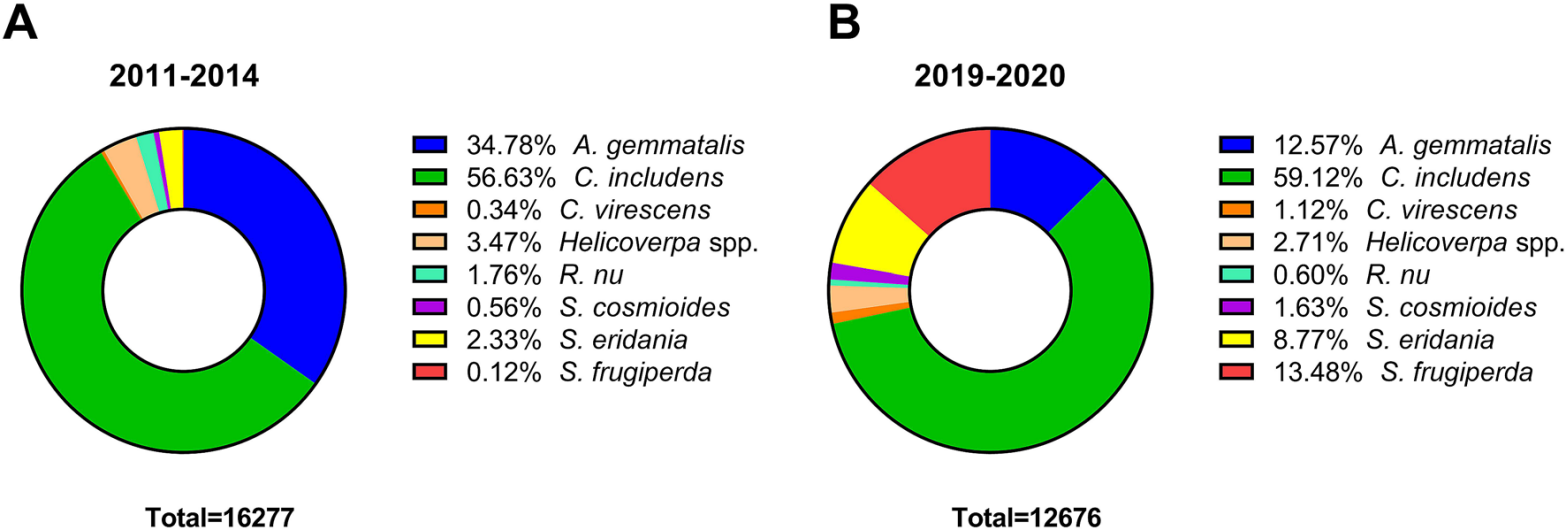
Lepidopteran species composition of sampled larvae from non-*Bt* fields during (**A**) pre-commercial (2011–2014) and (**B**) post-commercial Cry1Ac soybean (2019 and 2020) cropping seasons.

The percentage of each pest species relative to total larvae sampled in commercial (non-*Bt*) soybean fields prior to the commercialization of Cry1Ac soybean (2011 to 2014 cropping seasons; hereafter “pre-commercial period”) and total larvae sampled in commercial non-*Bt* soybean fields during the post-commercial period of Cry1Ac soybean (2019 and 2020 cropping seasons; hereafter “post-commercial period”) is shown in Fig. 2. Of the 16,277 lepidopteran larvae sampled in non-*Bt* fields during the pre-commercial period, more than 90% were *C. includens* and *A. gemmatalis* (Fig. 2A). The importance of these two species in non-*Bt* soybean fields was maintained during the post-commercial period, in which *C. includens* and *A. gemmatalis* represented more than 70% of 12,676 insects sampled (Fig. 2B). Small numbers of other species such *Helicoverpa* spp., *R. nu* and *C. virescens* were present, together representing less than 6% and less than 5% of the total samples during the pre- and post-commercial periods, respectively. *Spodoptera* species, represented mostly by *S. frugiperda, S eridania* and *S. cosmioides*, also were found in low numbers during the pre-commercial period. Representing only 3% of 12,676 insects sampled during the pre-commercial period, *S. eridania* was the predominant species sampled. However, *Spodoptera* species represented more than 23% of the total insects sampled in non-*Bt* soybean fields during the post-commercial period (Fig. 2B).

**Figure 3:**
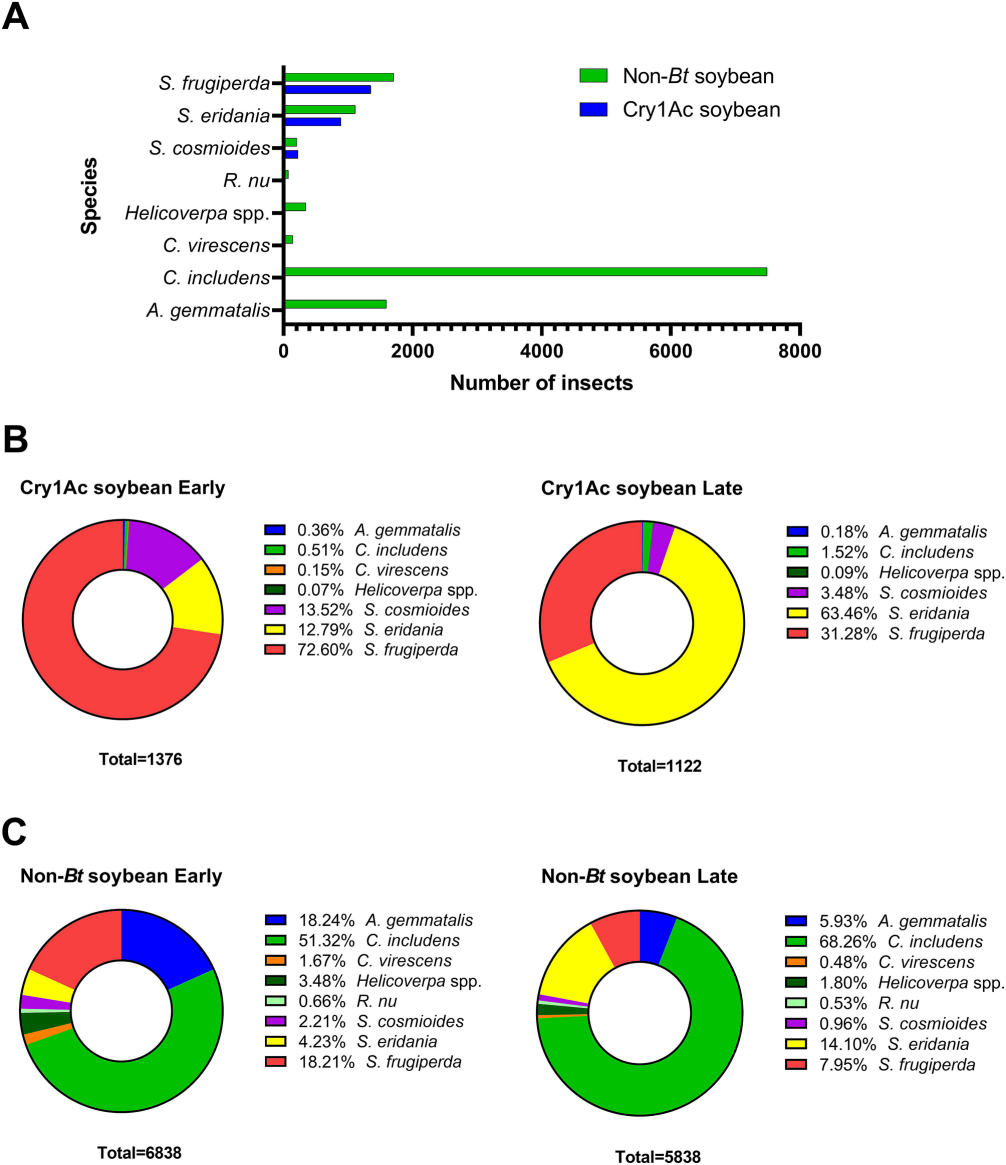
Lepidopteran species composition of sampled larvae from Cry1Ac soybean and non-*Bt* fields in the 2019 and 2020 cropping seasons. (**A**) Total number of lepidopteran larvae sampled in Cry1Ac soybean and non-*Bt* soybean fields. (**B**) Larvae species composition from Cry1Ac soybean fields in early and late stage. (**C**) Larvae species composition from non-*Bt* soybean fields in early and late stage.

The absolute numbers of *S. cosmioides*, *S. eridania* and *S. frugiperda* found in Cry1Ac and non-*Bt* soybean were similar (Fig. 3A). Of the 1,376 and 1,122 total insects sampled in Cry1Ac soybean at the early and late reproductive stages, respectively, more than 98% were *S. cosmioides*, *S. eridania* and *S. frugiperda* (Fig. 3B). More *S. frugiperda* were observed at the early reproductive stage and more *S. eridania* at the late reproductive stage in both soybean types (Fig. 3B). For non-*Bt* soybean, 12,676 larvae were identified, of which 51.32% and 68.26% were *C. includens* at the early and late reproductive stages, respectively (Fig. 3C). *S. frugiperda* and *A. gemmatalis* represented similar percentages of the total insects from non-*Bt* soybean at the early reproductive stage (18.21% and 18.24%, respectively) and late reproductive stage (7.95% and 5.93%, respectively) (Fig. 3C).

**Figure 4:**
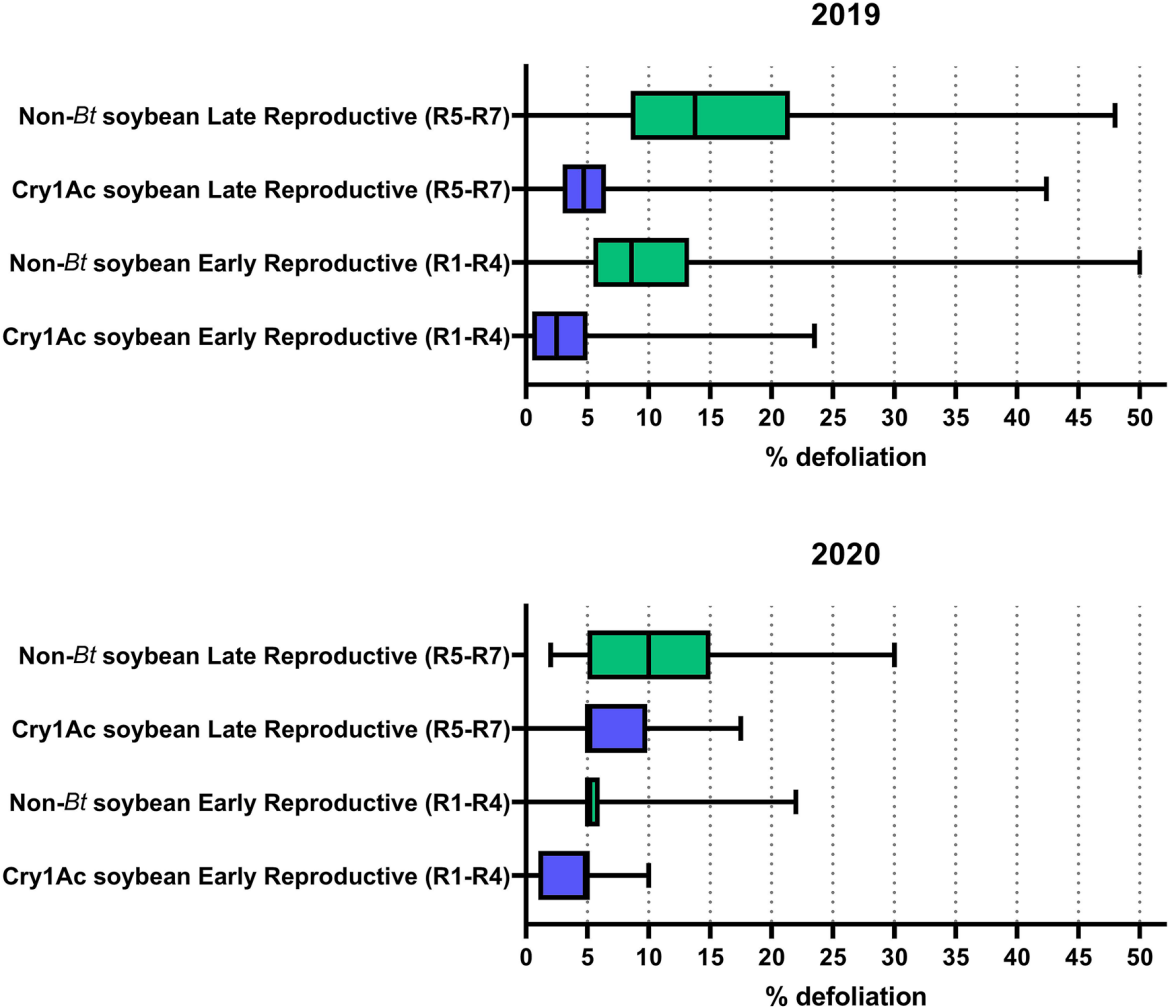
Defoliation in Cry1Ac soybean and non-*Bt* soybean fields in the 2019 and 2020 seasons. The middle vertical line within each box represents the median; the left and right edges of the boxes represent the 25th and 75th percentiles, respectively.

The median percentage defoliation in Cry1Ac soybean fields was lower than in non-*Bt* soybean fields in all but one comparison. For 2019, the Cry1Ac soybean median defoliation was 2.5% and 4.7% at the early and late reproductive stages, respectively, whereas median defoliation in non-*Bt* soybean fields was 8.6% and 13.7% at the early and late reproductive stages, respectively (Fig. 4). The 75th percentiles for defoliation in Cry1Ac soybean were 5% and 6.5% and for non-*Bt* soybean were 13.3% and 21.5% at the early and late reproductive stages, respectively (Fig. 4). In 2020, median defoliation in both Cry1Ac soybean and non-*Bt* soybean was 5% at the early reproductive stage, and 5% and 10%, respectively, at the late reproductive stage (Fig. 4). The 75th percentiles for defoliation at the late reproductive stage were 9.8% and 15% for Cry1Ac soybean and non-*Bt* soybean, respectively (Fig. 4).

### Comparison of pest abundance on Cry1Ac soybean and non-*Bt* soybean

Larval abundance in Cry1Ac soybean fields relative to non-*Bt* soybean fields in the 2019 and 2020 seasons, and 95% confidence intervals, are presented in Table 1. Based on these analyses, Cry1Ac soybean provided high control (relative abundance < 0.02) of *A. gemmatalis, C. includens, C. virescens* and *Helicoverpa* spp*.*; minimal control of *S. eridania* and *S. frugiperda*; and no control of *S. cosmioides* (Table 1). *R. nu* larvae were rare in samples from both non-*Bt* and Cry1Ac soybean fields. The relative Cry1Ac soybean abundance values were similar among early and late reproductive stages within each species, with the exception that *S. cosmioides* was more prevalent at the early reproductive stage (Table 1).

**Table 1:** Relative abundance of lepidopteran larvae in Cry1Ac soybean fields combined across the 2019 and 2020 seasons.

Species	Relative abundance^1^	LCL^2^	UCL^2^
**Early reproductive stage (R1–R4)**			
*A. gemmatalis*	0.004	0.002	0.01
*C. includens*	0.002	0.001	0.004
*C. virescens*	0.018	0.004	0.071
*Helicoverpa* spp.	0.004	0.001	0.03
*R. nu*	0	0	Inf
*S. cosmioides*	1.231	0.993	1.526
*S. eridania*	0.609	0.505	0.734
*S. frugiperda*	0.802	0.738	0.872
**Late reproductive stage (R5–R7)**			
*A. gemmatalis*	0.006	0.001	0.023
*C. includens*	0.004	0.003	0.007
*C. virescens*	0	0	Inf
*Helicoverpa* spp.	0.01	0.001	0.068
*R. nu*	0	0	Inf
*S. cosmioides*	0.697	0.463	1.048
*S. eridania*	0.865	0.783	0.957
*S. frugiperda*	0.758	0.66	0.871

^1^Relative abundance in Cry1Ac soybean vs non-*Bt* soybean.

^2^*LCL* lower confidence limit; *UCL* upper confidence limit (95% confidence interval).

### Pest species contributions to defoliation

Table 2 gives regression coefficient estimates for early and late reproductive stage non-*Bt* and Cry1Ac soybean. As described earlier, these coefficients represent the percentage increase in defoliation caused by each larva of a species. In non-*Bt* soybean fields, *A. gemmatalis*, *C. includens, Helicoverpa* spp., *S. cosmioides, S. eridania* and *S. frugiperda* contributed to defoliation at both early and late reproductive stages (P < 0.05) (Table 2). The highest coefficients observed for non-*Bt* soybean were for *S. cosmioides*: 1.385 and 2.136 for early and late reproductive stages, respectively (Table 2). Comparing *S. cosmioides* with *S. frugiperda* on early-reproductive-stage soybean, for example, the estimated coefficients were 1.385 and 0.245, respectively, indicating that an individual *S. cosmioides* larva caused 1.385/0.245 = 5.65 times the damage caused by an individual *S. frugiperda* larva. By that same logic, *S. cosmioides* caused 1.7 to 6.7 times the damage per larva caused by *A. gemmatalis*, *C. includens, Helicoverpa* spp*.* and *S. eridania* at the early reproductive stage. At the late reproductive stage, *S. cosmioides* caused 1.9 to 4.3 times the damage per larva caused by *A. gemmatalis*, *C. includens, Helicoverpa* spp., *S. eridania* and *S. frugiperda*.

**Table 2:** Relative contribution of lepidopteran species to early- and late-reproductive-stage defoliation in soybean fields, combined across the 2019 and 2020 seasons.

Technology	Species	Estimate^1^	Std. Error	t value	P( >|t|)
Non-*Bt* soybean	**Early reproductive stage (R1–R4)**				
	*A. gemmatalis*	0.204	0.026	7.748	< 0.0001
	*C. includens*	0.376	0.023	16.085	< 0.0001
	*C. virescens*	0.139	0.228	0.611	0.541
	*Helicoverpa* spp.	0.304	0.118	2.565	0.011
	*R. nu*	0.119	0.345	0.347	0.729
	*S. cosmioides*	1.385	0.246	5.632	< 0.0001
	*S. eridania*	0.81	0.139	5.824	< 0.0001
	*S. frugiperda*	0.245	0.055	4.452	< 0.0001
	**Late reproductive stage (R5–R7)**				
	*A. gemmatalis*	0.699	0.142	4.93	< 0.0001
	*C. includens*	0.649	0.044	14.717	< 0.0001
	*C. virescens*	0.718	0.656	1.095	0.274
	*Helicoverpa* spp.	1.118	0.498	2.247	0.025
	*R. nu*	0.419	1.129	0.371	0.711
	*S. cosmioides*	2.136	0.642	3.326	0.001
	*S. eridania*	0.501	0.101	4.95	< 0.0001
	*S. frugiperda*	0.619	0.19	3.259	0.001
Cry1Ac soybean	**Early reproductive stage (R1–R4)**				
	*A. gemmatalis*	0.643	1.715	0.375	0.708
	*C. includens*	0.125	1.53	0.081	0.935
	*C. virescens*	2.431	1.954	1.245	0.214
	*Helicoverpa* spp.	− 1.147	3.967	− 0.289	0.773
	*S. cosmioides*	0.623	0.092	6.797	< 0.0001
	*S. eridania*	0.309	0.077	3.99	< 0.0001
	*S. frugiperda*	0.137	0.027	5.162	< 0.0001
	**Late reproductive stage (R5–R7)**				
	*A. gemmatalis*	4.899	4.71	1.04	0.299
	*C. includens*	1.157	0.918	1.261	0.208
	*Helicoverpa* spp.	7	6.66	1.051	0.294
	*S. cosmioides*	2.121	0.712	2.978	0.003
	*S. eridania*	0.425	0.054	7.812	< 0.0001
	*S. frugiperda*	0.778	0.134	5.789	< 0.0001

^1^Estimate of the regression coefficient for a given species, which can be interpreted as the percentage increase in defoliation for each individual larva present.

For Cry1Ac soybean fields, only *S. cosmioides, S. eridania* and *S. frugiperda* contributed to defoliation (P < 0.05) (Table 2). The other species were controlled by Cry1Ac soybean, as described in the previous section. *S. cosmioides* had the highest coefficients in Cry1Ac soybean: 0.623 and 2.121 for early and late reproductive stages, respectively. Each *S. cosmioides* larva caused 2.0 and 4.5 times the damage at the early reproductive stage and 4.9 and 2.7 times the damage at the late reproductive stage caused by individual *S. eridania* and *S. frugiperda* larvae, respectively.

### Geographic variation in soybean pest abundance

**Figure 5:**
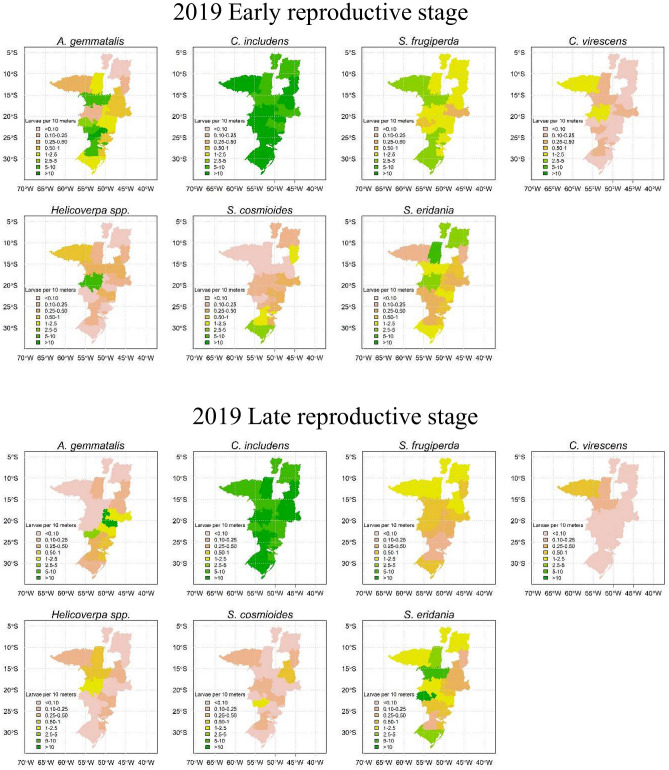
Pest abundance (larvae per 10 m of beat sheet) in non-*Bt* soybean by geographic region in 2019 season.

**Figure 6:**
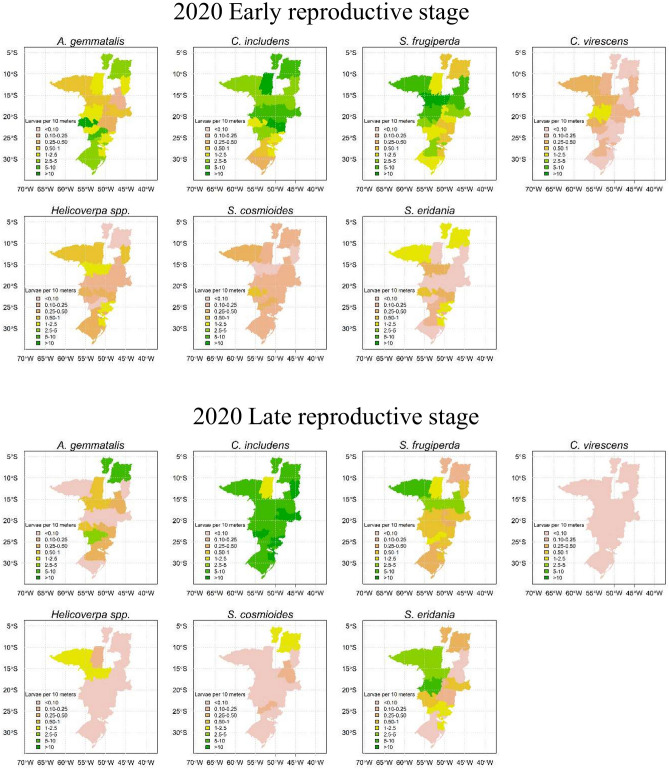
Pest abundance (larvae per 10 m of beat sheet) in non-*Bt* soybean by geographic region in 2020 season.

Visualization of pest abundance in non-*Bt* soybean by species in Figs. 5 and 6 shows that *C. includens* was present at high levels in all of the soybean-producing regions sampled in Brazil. *A. gemmatalis* and *S. eridania* abundance varied among seasons and growth stages but both species were often present at high levels. *S. frugiperda* abundance was lower in 2019 than in 2020, when there was high infestation in northern regions (Figs. 5 and 6). Abundances of *C. virescens*, *Helicoverpa* spp. and *S. cosmioides* were lower across the regions evaluated when compared to *C. includens*, *A. gemmatalis, S. eridania* and *S. frugiperda* (Figs. 5 and 6).

### Insecticide spray usage on soybeans fields in Brazil

**Figure 7:**
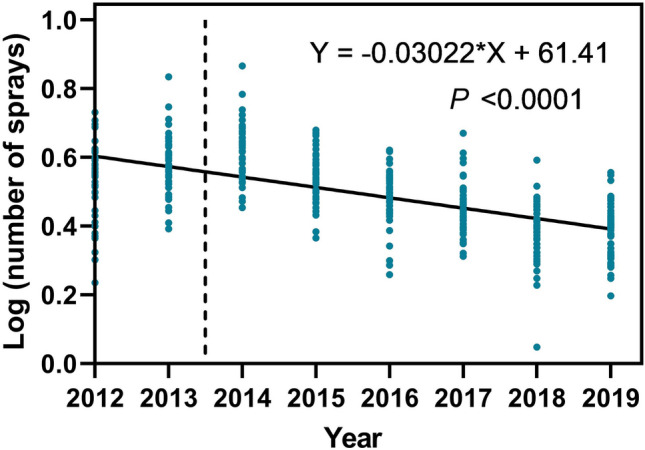
Number of insecticide sprays used to manage lepidopteran larvae across mesoregions and crop seasons in Brazil. Dashed line represents the start of commercial planting of Cry1Ac soybean in Brazil. Data on use of insecticide sprays to manage lepidopteran larvae across mesoregions for the 2013 to 2019 cropping seasons were obtained from the AMIS Kleffmann Group database (2013–2018) and BIP Spark (2019).

The number of sprays for management of lepidopteran larvae over mesoregions decreased from an average of 3.5 in 2012 to 2.45 in 2019 cropping season (F = 182.5, df = 1,354, R^2^ = 0.34, P < 0.0001) (Fig. 7).

## Discussion

This work presents the most extensive geographic assessment of lepidopteran pests on Brazil's soybean fields that covers more than 35 million hectares. *C. includens* was the main lepidopteran species occurring in non-*Bt* soybean (RR) fields in our study, being present in all the regions evaluated. This species was considered a secondary pest of soybean until the early 2000s. Its relevance in soybean likely increased because of changes in cultivation systems (i.e., no-till and cultivation of multiple crop and non-crop hosts of this species) and a decline in the adoption of IPM practices^7,37^. Both cotton and soybean have been documented as suitable hosts of *C. includens*^38–41^. The large increase in soybean cultivation area in Brazil seems to be a particularly important component in *C. includens* adaptation^31^. Soybean farms now are predominant in the agricultural landscape, narrowing the host plant availability to *C. includens* in some regions^31^. Increases in cultivation of other crop and non-crop hosts of *C. includens* also may have created a “green bridge” favoring the growth and spread of populations^7,31^. This likely increased the selective pressure of insecticides and *Bt* soybean plants, leading to higher resistance risk for these control tactics. *C. includens* prefers to feed on the lower and mid canopies of soybean plants, making it difficult to manage with insecticide sprays in the first place^42^. The resistance of *C. includens* populations to pyrethroids and chitin synthesis inhibitors has further contributed to the increased prevalence of this pest^43,44^. However, even under this high-resistance-risk scenario, our data showed that Cry1Ac soybean continues to be effective at controlling this pest. The near-high-dose level of the Cry1Ac *Bt* soybean MON 87701 × MON 89788 against *C. includens*^12^ and the low initial resistance allele frequency^45^ in *C. includens* have been key to managing the risk of *Bt* resistance in this pest.

*A. gemmatalis* was recognized as a major defoliating insect associated with soybean fields in Brazil, requiring an average of 2 insecticide applications every season^46^. Our results showed that the abundance of *A. gemmatalis* was lower than that of *C. includens* before and after the commercial launch of Cry1Ac soybean (2011–2014 and 2019–2020 cropping seasons). In the 2011 to 2014 sampling, *A. gemmatalis* was the second most abundant pest after *C. includens,* confirming that these two were the major pests of soybean in the early part of the decade. *A. gemmatalis* feeds primarily on leguminous plants (at least 34 species within Fabaceae family) and on only three other families (Begoniaceae, Poaceae and Malvaceae), with five species in these families serving as larval hosts^47,48^. This relatively narrow host range, in combination with the high efficacy of Cry1Ac soybean against *A. gemmatalis*^12^, may have contributed to reduced abundance of this species in Brazil. Our analysis showed that *A. gemmatalis* made a significant contribution to defoliation and is widely distributed in non-*Bt* soybean fields, so it is important to monitor for this species. In contrast, the low relative abundance of *A. gemmatalis* in Cry1Ac soybean showed that the pest is being effectively controlled by this technology in Brazil eight years after commercial launch.

*Helicoverpa* spp. was found at relatively low abundance. Within the *Helicoverpa* species found in Brazil, *H. armigera* are prevalent on dicotyledonous hosts such as soybean and cotton and *H. zea* on maize^49–52^. Therefore, most of the *Helicoverpa* spp. larvae in our collections are likely to be *H. armigera*. Although this species was first reported causing damage in soybean in Brazil^53^, the suitability of cotton as a host plant seems to be higher than soybean, as evidenced by higher larval viability and net reproductive rate^52,54^. The broad cultivation of row crops (e.g. soybean, cotton and maize) and availability of non-crop hosts across Brazil throughout the year, in combination with the high polyphagia of *H. armigera*, may be shaping the dynamics of this pest^5,52^. The relatively low abundance of this pest in soybean in Brazil also may be related to the broad adoption of Cry1Ac soybean, which is highly efficacious against this pest^14,52^.

The prevalence of *S. frugiperda* in non-*Bt* soybean was higher in 2019–2020 than in 2011–2014, increasing from 0.12% to more than 13%. *S. frugiperda* is a major pest of maize and cotton^30,55–57^, but recently has been also reported as a pest of soybean in Brazil^6,58,59^. The occurrence of *S. frugiperda* on soybean is favored by its ability to develop on several host plants^60^, high dispersal and migratory capacity^61,62^, high reproductive potential^63^, adaptation to Brazilian crop systems with availability of suitable hosts throughout the year^30^, and resistance to several classes of insecticides^64–69^. Although soybean plants produce proteinase inhibitors, *S. frugiperda* can adapt by altering the composition of proteolytic enzymes in the midgut^70^. *S. frugiperda* also expresses detoxification gene families that enable rapid response to plant secondary metabolites^71^. The abundance of green plant material provided by winter cover crops such as millet, which are highly suitable for *S. frugiperda*^57^, can also contribute to keeping populations of this pest at reasonably high levels throughout the year, creating a “green bridge” enabling dispersal and/or migration among hosts. Another factor that may be influencing *S. frugiperda* population growth and increasing its occurrence in soybean is the recent increase in winter maize area, where maize is rotated with soybean, and decrease in summer maize acreage in Brazil^5^. Currently, winter maize is planted on more than 13 million hectares, representing most of the maize planted in Brazil; in contrast, summer maize represented 4.3 million hectares in 2020^5^. The removal of a significant number of maize plants from the landscape during the summer season may have triggered *S. frugiperda* to more frequently explore and colonize other suboptimal but readily available hosts such as soybean. Our analyses indicate that *S. frugiperda* could contribute to defoliation in soybean fields, though its capacity to defoliate soybean is lower than some other *Spodoptera* species.

*S. eridania* was more abundant than *S. frugiperda* during the late reproductive stages of soybean. *S. eridania* is also a polyphagous pest, reported to be capable of feeding on 202 host plant species^72^. Compared to cotton, soybean is a less suitable host for *S. eridania*, leading to lower pupal survivorship when consumed exclusively^73^. However, in the soybean–cotton farming system in the Cerrado region of Brazil (in the Central-West of the country), this pest may be of greater importance because it can find a continuous source of food in these two crops^73^. Sampling of lepidopteran larvae from soybeans at four locations in Mato Grosso do Sul State in 2011/12 showed that *Spodoptera* species represented about 10% of lepidopteran larvae in the samples^74^. Another study at one location in 2015/16 showed that *Spodoptera* accounted for 5% of the total lepidopteran larval sample: among these larvae, more than 50% were *S. eridania*^75^. In addition to feeding on leaves, *Spodoptera* species can feed on soybean pods^6^, which may have contributed to the higher density of *S. eridania* observed at the late reproductive stage in our collections.

*S. cosmioides* was at lower abundance than the other two *Spodoptera* species mentioned above, but its capacity to defoliate the soybean crop was greater than that of any other species in our collections. Its high capacity to cause damage has been demonstrated under laboratory conditions. For example, *S. cosmioides* was able to defoliate nearly twice the area defoliated by *A. gemmatalis*, *S. eridania* or *S. frugiperda*^6^. *S. cosmioides* is also a polyphagous pest capable of feeding on 126 plant species^76^. Soybean and cotton are conducive to development of this species, but maize does not allow its larval development^77,78^. Soybean and cotton also are preferred hosts for oviposition of the species when compared to oats, wheat and maize^78^. Therefore, the monitoring of this pest in soybean and cotton fields is important to prevent yield loss due to significant defoliation or pod damage.

The *Spodoptera* species are not controlled by Cry1Ac soybean, so their presence is expected in both Cry1Ac soybean and non-*Bt* soybean fields^15^. Larvae of *Spodoptera* species predominated in Cry1Ac soybean fields in our study, and the numbers of these three species were similar between Cry1Ac soybean and non-*Bt* soybean fields. Therefore, any differences in lepidopteran control tactics adopted by growers in Cry1Ac soybean and non-*Bt* soybean fields have not resulted in an increase in density of these *Spodoptera* species on Cry1Ac soybean relative to non-*Bt* soybean fields.

Abundance of both *C. virescens* and *R. nu* was low in our samples from the 2019 and 2020 seasons. Both species are considered pests of soybeans in Brazil^33^. Combined, these species represented less than 2.3% and 1.0% of the samples at the early and late reproductive stages in non-*Bt* soybean, respectively. Low abundance of these species was also observed in the 2011–2014 samples. *C. virescens* is a major pest in cotton and used to be observed attacking soybean in the central region of Brazil^79^. Cry1Ac soybean meets the high-dose concept for *C. virescens*^13^ and continues to provide effective control according to our results. In the USA, this pest is also considered an important pest of cotton, and the high adoption of *Bt* cotton in the USA may have reduced its abundance over large areas^80^. In Brazil, high adoption of both *Bt* cotton and Cry1Ac soybean could also be influencing the abundance of *C. virescens*. *R. nu* occurs predominantly in southern South America, and this pest is an important defoliator of soybean in Argentina^81^. *R. nu* is more adapted to subtropical and temperate regions than to tropical regions and is favored by higher latitudes and altitudes^41^. However, this pest has been reported from southern (Rio Grande do Sul and Paraná) to central regions of Brazil (Distrito Federal)^75,82–84^.

Despite fluctuations in lepidopteran pest abundance across regions and cropping seasons, *C. includens* and *A. gemmatalis* continue to be the main lepidopteran pests on non-*Bt* soybean in Brazil. The absence or very low density of these two species and reduced levels of defoliation on *Bt* soybean observed across regions and seasons indicate that Cry1Ac soybean still provides effective protection against these species. A significant reduction in the number of insecticide sprays to manage lepidopteran larvae has occurred over mesoregions and crop seasons, indicating that increasing adoption of Cry1Ac soybean has effective managed and apparently suppressed *C. includens* and *A. gemmatalis* populations across soybean-growing regions. Assessing the environmental impact of this reduction in lepidopteran sprays would be worthwhile. Suppression of target pests after a long period of use of *Bt* technologies has been documented in *P. gossypiella*, *O. nubilalis* and *H. zea* in the USA^18,19,21^ and *H. armigera* in China^20^. However, *Spodoptera* species are not controlled by Cry1Ac soybean^15^, and consequently they can be found on both Cry1Ac and non-*Bt* soybean. The occurrence of *Spodoptera* species, which were historically considered as secondary pests of soybeans in Brazil, on Cry1Ac and non-*Bt* soybeans may be associated with the high efficacy of the Cry1Ac soybean against target species (i.e., *C. includens* and *A. gemmatalis*) and the resulting reduction in insecticide use in soybeans fields in Brazil (Fig. 7). The challenge posed by secondary pests such as *Spodoptera* species highlights the need to develop *Bt* soybean technologies with novel modes of action^59,85,86^. When available, *Bt* soybean technologies with diverse modes of action will enhance pest management systems for soybean in Brazil.

It is also important to emphasize that the planting of refuge is crucial to the management of *Bt* crop pests. In Brazil, soybean, maize and cotton are planted simultaneously or in succession within the Cerrado landscape^30^. The proteins used in *Bt* soybean, maize and cotton overlap to a large degree and several of the most important target pests feed on two or all three of these crops e.g., *S. frugiperda*, *H. armigera* and *C. includens*, as discussed herein*.* Cross-crop resistance is a threat to their management. Indeed, resistance of *S. frugiperda* to *Bt* maize is already affecting the efficacy of *Bt* soybean and cotton due to cross resistance resulting from shared or similar *Bt* proteins among technologies^59,87^. Therefore, adherence to refuge recommendations for *Bt* maize, cotton and soybean is necessary to enhance durability of current and future *Bt* technologies in this multi-crop agroecosystem.

Overall, our study provides a large-scale assessment of Cry1Ac soybean field efficacy and demonstrates that its pest control benefits are being sustained. Cry1Ac soybean has provided Brazilian farmers with eight years of consistent protection against damage from the primary lepidopteran soybean pests (*C. includens* and *A. gemmatalis*). However, Cry1Ac soybean needs to be viewed as one tool within the pest management toolbox and should be integrated with other effective control tactics.
